# Combination of G-CSF and AMD3100 Improves the Anti-inflammatory Effect of Mesenchymal Stem Cells on Inducing M2 Polarization of Macrophages Through NF-κB-IL1RA Signaling Pathway

**DOI:** 10.3389/fphar.2019.00579

**Published:** 2019-05-28

**Authors:** Long Chen, Qian Zhang, Qin-Hua Chen, Feng-Yin Ran, Li-Mei Yu, Xiu Liu, Qiang Fu, Gong-Yu Song, Jun-Ming Tang, Tao Zhang

**Affiliations:** ^1^Key Laboratory of Cell Engineering of Guizhou Province, Affiliated Hospital of Zunyi Medical University, Zunyi, China; ^2^Experimental Medical Center, Dongfeng Hospital, Hubei University of Medicine, Shiyan, China; ^3^Department of Human Anatomy, Zunyi Medical University, Zunyi, China; ^4^Institute of Clinical Medicine, Renming Hospital, Hubei University of Medicine, Shiyan, China; ^5^Institute of Biomedicine and Key Lab of Human Embryonic Stem Cell of Hubei Province, Hubei University of Medicine, Shiyan, China

**Keywords:** peripheral blood-derived mesenchymal stem cells, anti-inflammatory, macrophage, polarization, IL1rn, NF-κB

## Abstract

Mobilized peripheral blood-derived mesenchymal stem cells (PB-MSCs) mainly derived from bone marrow-derived MSCs (BM-MSCs) exert a similar anti-inflammatory effect. However, the mechanism of anti-inflammatory effect of mobilized PB-MSCs by a combination of G-CSF and AMD3100 remains unclear. Cultured rat PB-MSCs mobilized by G-CSF/AMD3100 have shown typical surface markers and potential for multiple differentiations, similar to non-mobilized BM-MSCs. In a co-culture system, rat M0-type macrophages co-cultured with PB-MSCs have shown higher expression of M2 markers including CD206, Arg-1, IL-10, and CCL-22 than BM-MSCs, indicating that PB-MSCs induced greater M0 polarization to M2. Furthermore, compared with BM-MSCs, PB-MSCs in a co-culture system with lipopolysaccharide-induced M1-type macrophages more efficiently promoted M1 polarization to M2, accompanied by increasing expression of CD206, Arg-1, IL-10, and CCL-22 while decreasing expression of M1 markers including iNOS, TNF-α, IL-1β and IL-6, indicating that PB-MSCs triggered greater M1 polarization to M2. Subsequently, polymerase chain reaction arrays showed higher expressions of both IL1rn and Tnfrsf11b in PB-MSCs versus BM-MSCs. In response to an inflammatory niche, such as TNF-α, PB-MSCs have shown higher expression and release of IL1RA, causing greater M2 polarization of macrophages, and the special effects may be almost entirely abolished through the neutralization antibody of IL1RA. Mechanistic studies determined that PB-MSCs showed higher levels NF-κBp65 and NF-κBp-p65 than BM-MSCs, which could be obviously enhanced by TNF-α. And the increased IL1RA expression by TNF-α in PB-MSCs could be markedly canceled by an NF-κB inhibitor PDTC. Interestingly, mimicking the mobilized PB-MSCs by a combination of G-CSF and AMD3100 *in vivo*, BM-MSCs were treated with G-CSF and/or AMD3100 *in vitro*, showing the increased expressions of NF-κBp65 and IL1RA, which could be prominently abolished by PDTC. Therefore, targeting IL1rn, gene modification or drug intervention for MSCs may provide a novel therapeutic strategy for human diseases, especially inflammatory diseases.

## Introduction

Inflammation is a common cause of numerous human diseases, such as heart disease, diabetes mellitus, Alzheimer’s disease, hepatitis, acute lung injury, arthritis, and acute pancreatitis. Indeed, macrophage activation and phenotypic switch of macrophages (M0) from a pro-inflammatory phenotype (M1) or an anti-inflammatory phenotype (M2) may play a crucial role in the inflammatory response and numerous associated diseases (Tabas and Bornfeldt, 2016). M1 is characterized by increased levels of iNOS, TNFα, IL-1β, IL-12, and IL-6, and promotes the inflammatory action by activating T-cell priming and recruitment into the inflammatory site (Zhou et al., 2014). In contrast, M2 macrophages function as anti-inflammatory mediators to repair the injured tissues by releasing anti-inflammatory cytokines, such as CCL22, IL-10, IL-13, and IL-33 (Jiang et al., 2014). Therefore, a novel target to induce the macrophage phenotypic switch toward M2 is necessary to diminish the inflammatory response.

The interleukin-1 (IL-1) family is a key regulation mechanism of monocyte–macrophage differentiation through a complex network of transcription factors in numerous inflammatory processes (Ramadas et al., 2011; Chamberlain et al., 2013; Grebe and Latz, 2013). Of note, IL-1α, IL-1β, and IL-1RA are the most widely studied members of the IL-1 family, and their respective biological activities are pivotal in inflammatory mechanisms for the activation of macrophages (Dinarello, 1996). Both IL-1α and IL-1β interact with the IL-1 receptor 1 (IL-1R1) and recruit the IL-1 receptor accessory protein (IL-1R3, formerly termed IL-1RAcP) to induce a downstream signal via several inflammatory kinases, such as MAPK-P38, ERK, JNK, AKT, and NF-κB, leading to transcription of inflammatory cytokines (Huaux et al., 2015; Sakai et al., 2015; El et al., 2018; Maruyama et al., 2019). On the other hand, IL-1RA exerts its anti-inflammatory effects by binding to the IL-1R1 and specifically inhibiting IL-1 signaling (Hannum et al., 1990). Therefore, increasing the levels of the members of the IL-1 family (IL-1RA) may be beneficial in inducing the macrophage phenotypic switch toward M2.

Mesenchymal stromal cells (MSCs), such as adipocytes, chondroblasts, and osteoblasts (Wong, 2011), which can differentiate into multiple types of cells, play important roles in immune and inflammatory modulation (Machado et al., 2013; Wang et al., 2014). Bone marrow-derived MSCs (BM-MSCs), one of the multiple sources of MSCs, induce either M0 or M1 macrophage polarization toward M2 through direct contact between cells (Kim and Hematti, 2009; Nemeth et al., 2009; Chiossone et al., 2016; Luz-Crawford et al., 2016), providing a potential therapeutic strategy for the treatment of inflammatory diseases (Wang et al., 2016). Of note, different original MSCs from the bone marrow, peripheral blood, or other tissues with similar surface markers have shown specific differences in gene expression profiles related to inflammation (Machado et al., 2013; Zhou et al., 2013). These findings indicate that different MSCs may contribute differently to inflammatory regulation (Zhou et al., 2013; Wang et al., 2014).

NF-κB, a key transcription factor regulating the production of cytokines by MSCs, may be activated by pro-inflammatory cytokines, such as interleukin 1 (IL-1β) and tumor necrosis factor α (TNFα), enhancing the paracrine effects of MSCs (Luz-Crawford et al., 2016). However, the involvement of peripheral blood-derived MSCs (PB-MSCs) in the macrophage phenotypic switch and anti-inflammatory effects remains to be determined.

The aim of this study was to investigate the role and mechanism of PB-MSCs mobilized by G-CSF/AMD3100 in the regulation of macrophage polarization, versus BM-MSCs.

## Materials and Methods

### Mobilization, Harvest, and Culture of Both PB-MSCs and BM-MSCs

Adult male Sprague–Dawley rats (weight: 250–300 g) were purchased from the Laboratory Animal Centre, Hubei University of Medicine (SCXK 2011-0008, Shiyan, China), and housed in a specific-pathogen-free laboratory animal room. Food (130–240 g weekly) and water (75 mL daily day) were supplied to each rat. The rats were raised for 7 days for acclimatization. All animal experiments conformed to the protocol approved by the Hubei University of Medicine Health Network Animal Care Committee. The rats were initially treated with G-CSF (100 μg/kg; Qilu Pharmaceutical Co., Ltd., Jinan, China) for 5 days via intraperitoneal injection once daily. After 6 days, the CXCR4 antagonist AMD3100 (5 mg/kg via intraperitoneal injection; Selleck Chemicals, Houston, TX, United States) was used to supplement stem cell mobilization. One hour after the treatment with AMD3100, 4–5 mL of peripheral blood was harvested from rats under anesthesia through the abdominal aorta using a tube with heparin (300 U/mL).

The blood samples were diluted 1:1 with phosphate-buffered saline (PBS). Mononuclear cells were isolated using density gradient centrifugation Lymphoprep Ficoll 1.077 g/mL (Haoyang Biological Manufacture, Tianjing, China), according to the instructions provided by the supplier. After centrifugation, the cells at the gradient interface were collected, washed with PBS, passed through a 100-μm mesh filter, and resuspended in a complete Dulbecco’s modified Eagle’s medium (DMEM; Gibco, Waltham, MA, United States) containing 15% fetal bovine serum, 2.2 g NaHCO_3_, 100 U/mL penicillin, 100 mg/mL streptomycin, 25 ng/mL amphotericin B, and 2 mM L-glutamine at a density of 2.5 × 10^6^ cells/mL. Finally, the cells were incubated at 37°C with 5% CO_2_. After 3 days, the non-adherent cells were removed, and a fresh complete medium was added. The medium was changed every 3 days. At 10 days, cells were digested using 0.25% trypsin/1 mM EDTA and harvested for passage. PB-MSCs passaged thrice were used for the following experiment.

To obtain and culture BM-MSCs, the lower limbs of the rats with or without administration of G-CSF and AMD3100 were collected under sterile conditions. The muscles and connective tissues were removed from both the tibia and the femur. Subsequently, BM-MSCs were harvested through slight flushing with PBS supplemented with 1% penicillin/streptomycin. The cell suspension was filtered through a 100-mm filter mesh to remove any bone spicules, muscles, and cell clumps. Mononuclear cells in the cell suspension were isolated through density gradient centrifugation and cultured as previously described (Fu et al., 2012). BM-MSCs passaged thrice were used for the following experiment.

### Identification of Surface Markers in PB-MSCs and BM-MSCs

Fluorescence-activated cell sorting analyses of surface markers in PB-MSCs and BM-MSCs were performed. Cell aliquots (1 × 10^6^ cells, passaged thrice) were trypsinized using 0.25% trypsin/EDTA, washed twice with PBS, suspended in 500 μL PBS, mixed with mouse antibodies against rat CD90 (1:100, eBioscience, San Diego, CA, United States), CD29 (1:100, Biolegend, San Diego, CA, United States), CD34 (1:50, Santa Cruz, Dallas, TX, United States), CD44 (1:100, Santa Cruz, Dallas, TX, United States), CD11b (1:100, Santa Cruz, Dallas, TX, United States), or CD45 (1:100, eBioscience, San Diego, CA, United States), and incubated on ice for 30 min. In addition, the samples were labeled with Alexa Fluor 488-conjugated donkey anti-mouse secondary antibody for 30 min. Homologous isotope IgG controls were included in each experiment to eliminate non-specific staining.

### Multi-Differentiation of PB-MSCs and BM-MSCs

The multi-differentiation potential of MSCs was induced as previously described (Yaochite et al., 2016). Briefly, adipogenesis was induced with 10% fetal calf serum-DMEM containing 16 μM biotin, 18 μM panthotenic acid, 100 μM ascorbic acid, 5 μg/mL insulin, 0.03 μM dexamethasone, 1 μg/mL transferrin, and 2 ng/mL triiodothyronine (T3) (Sigma–Aldrich, SaintQuentin Fallavier, France) for 21 days. Oil red O staining was performed to determine the adipogenic differentiation of MSCs.

For the induction of osteogenesis, MSCs were plated at a 1 × 10^5^ cells/cm^2^ density in DMEM supplemented with 10% fetal calf serum, 2 mM L-glutamine, and 50 μg/mL ascorbic acid. The medium was changed every 2 days for 21 days. Alizarin red S staining was performed to identify osteogenic differentiation of MSCs.

For chondrocyte induction, MSCs were plated at 1 × 10^5^ cells/cm^2^ in DMEM supplemented with 10% fetal calf serum, 100 × ITS (Sigma–Aldrich, St. Louis, MO, United States), 1 mM pyruvate, 0.17 mM ascorbate, 0.1 mM dexamethasone, 0.35 mM proline, and 10 ng/mL TGFβ. The medium was changed every 2 days for a total of 21 days. Alcian Blue staining was performed to determine the osteogenic differentiation of MSCs.

### Macrophage Culture and Polarization

For the generation of bone marrow-derived macrophages, bone marrow cells were isolated from the femur and tibia of mice, and cultured in 15-cm bacteriologic plastic Petri dishes in RPMI 1640 medium supplemented with 10% fetal bovine serum, 100 U/ml penicillin, 100 mg/ml streptomycin, 2 mM L-glutamine, 10 mM HEPES (Gibco, Waltham, MA, United States), and 20% L-929 cell conditioned medium (CM) for 6 days. These differentiated macrophages were harvested and plated overnight in six-well plates (2 × 10^6^ cells/well). In addition, the cells were polarized for 24 h using either the M1-type inducer lipopolysaccharide (100 ng/mL, L2630 Sigma–Aldrich, Louis, MO, United States) or the M2-type inducers IL-4 (20 ng/ml, PeproTech, Rocky Hill, NJ, United States) and IL-10 (10 ng/ml, R&D Systems, Minneapolis, MN, United States). Non-polarized macrophages were designated as M0 type. For the analysis of gene expression, real-time polymerase chain reaction (PCR) was performed 24 h after polarization of the macrophages.

### Co-culture of Macrophages With MSCs

For the establishment of the co-culture system of macrophages with MSCs using the Transwell system, macrophages (2 × 10^5^ cells/well) were pre-plated in six-well plates for 7 days and the Transwell chambers (0.4 μm pore size, Corning, Lowell, MA, United States) were placed in six-well plates. Subsequently, MSCs (2 × 10^5^ cells/well) suspended in RPMI 1640 complete medium for macrophages were added on the top of each Transwell. Finally, the co-culture system was placed in an incubator for 3 days at 37°C with 5% CO_2_.

### Intracellular Cytokine Staining

For the detection of cytokine expression levels within macrophages, macrophages were stimulated with the M1-type inducer lipopolysaccharide (100 ng/mL) for 24 h, or the M2-type inducers IL-10 (20 ng/mL) and IL-4 (20 ng/mL) for 24 h. A protein transport inhibitor cocktail (eBioscience, San Diego, CA, United States) was added to block the secretion of cytokines, and specific staining was performed using an intracellular fixation buffer and permeabilization buffer (eBioscience, San Diego, CA, United States) (Zhou et al., 2014). Briefly, the cells were stained with antibodies against surface markers for 15 min at room temperature. The cells were subsequently fixed, washed, and permeabilized. Cytokine-specific antibodies, such as antibodies against iNOS, TNFα, CD206, and Arg-1, were added, and the samples were incubated for 20 min at room temperature. Subsequently, the samples were washed and fixed with 1% paraformaldehyde in PBS for fluorescence-activated cell sorting assays as previously described.

### High-Throughput Quantitative PCR (HT-qPCR)

High-Throughput Quantitative PCR analysis was performed using an Applied Biosystems ViiATM 7 Real-Time PCR System (Thermo Fisher Scientific Inc., Waltham, MA, United States). Eighty-four validated primer sets were used to target inflammatory cytokines and receptors, such as chemokines, chemokine receptors, interleukins, interleukin receptors, other cytokines, and other cytokine receptors (the gene symbols are shown in Supplementary Table S1). The qPCR mixture (10 μL) consisted of 5 μL Roche FastStart Universal SYBR Green Master (2×) (Indianapolis, IN, United States), 0.75 μL of each primer (10 μM), 3 μL ddH_2_O, and 0.5 μL template (the concentration of DNA ranged between 28.5 and 255.0 ng/μL). All qPCR reactions were performed in triplicate. Denaturation was performed at 95°C for 10 min, followed by 40 cycles at 95°C for 30 s, and 30 s annealing at 60°C. A final melting curve was generated, ranging from 60 to 95°C, to determine the specificity of amplification. In this study, a threshold cycle (Ct) of 31 was used as the detection limit.

### Analysis of the Differential Gene Expression Profiles in PB-MSCs

Peripheral blood-derived mesenchymal stem cells or BM-MSCs RNA samples were obtained from the same animal (*n* = 6). Subsequently, mRNA was extracted from each sample and HT-qPCR was performed using a rat inflammatory Cytokines and Receptors RT^2^ Profiler PCR Array (Wcgene Biotechnology, Shanghai, China). Three arrays were used for each experimental group, and each sample was examined in triplicate. Differences in gene expression between PB-MSCs and BM-MSCs were considered significant at a fold change > 2.5 and *p* < 0.001. The expression profiles of 84 genes are listed in Supplementary Table S1.

The 23 differentially expressed genes were considered seed molecules from which we obtained direct and indirect protein–protein interactions using the STRING 9.0 database (Search Tool for the Retrieval of Interacting Genes). This database contains information regarding experimental and predicted interactions from varied sources based on their neighborhood, gene fusions, co-occurrence, co-expression, experiments, and literature mining. We constructed an extended network based on a high confidence score of 0.7. This implied that only interactions with a high level of confidence were extracted from the database and considered valid links for the protein–protein interaction network.

### Quantitative Real-Time PCR

Total cellular RNA was isolated from MSCs and macrophages using the Gene JET RNA Purification Kit (Thermo Fisher Scientific, Inc., Waltham, MA, United States) according to the instructions provided by the manufacturer. Total RNA was quantified via a spectrophotometer, and RNA integrity was assessed using 1% agarose gels. Approximately 1 mg of total RNA from each sample was synthesized to cDNA according to the instructions provided by the manufacturer, using a Revert Aid First-Strand cDNA Synthesis Kit (Thermo Fisher Scientific, Inc., Waltham, MA, United States). PCR was performed using the Fast Start Universal SYBR PCR Master Mix (Qiagen, Mannheim, Germany). Amplification was performed using the Rotor Gene 6000 Real-Time PCR System (Qiagen, Mannheim, Germany) with a two-step PCR protocol (preincubation for 10 min at 95°C, followed by 30 cycles at 95°C for 15 s and for 1 min at 60°C). The list of primer sequences is shown in Supplementary Table S2. Following normalization using GAPDH mRNA, the comparative threshold method (^ΔΔ^CT method) was used to perform the relative quantification of the samples (relative quantitation computer software; Applied Biosystems). Fold changes in gene expression were calculated using the equation 2^−ΔΔCT^.

### Western Blotting Analysis

Mesenchymal stem cells were lyzed in ice-cold lysis buffer (RIPA buffer, Millipore, Burlington, MA, United States) on ice. Protein quantification in cell lysates was performed using the Bradford (Bio-Rad, Hercules, CA, United States) assay. Equal amounts of proteins were separated by 10% sodium dodecyl sulfate-polyacrylamide gel electrophoresis, and transferred to a 0.22-μm polyvinylidene difluoride membrane. The membranes were blocked with 10% non-fat milk in TBS-Tween solution (0.05% Tween 20 in Tris-buffered saline), incubated overnight at 4°C with indicated primary antibody, and washed with TBS-Tween solution. Subsequently, the membranes were incubated with the appropriate horseradish peroxidase-conjugated secondary antibodies (1:10,000) for 2 h at room temperature, followed by washing with TBS-Tween solution. The immunoblots were developed using the Super Signal West Pico Chemiluminescent Substrate (Thermo Fisher Scientific Inc., Waltham, MA, United States) and a digital luminescent image analyzer Biospectrum 600 (UVP, Upland, CA, United States). Measurements were performed through densitometry using the ImageJ software (Copyright,1.48, NIH). The primary antibodies are shown in Supplementary Table S3.

### Preparation of CM

Peripheral blood-derived mesenchymal stem cells or BM-MSCs were grown until they reached 80–90% confluence, washed with PBS, and starved overnight in serum-free medium. To generate activated MSC CM (^TNFα^CM), cells were cultured for 24 h in either serum-free medium (LG-DMEM) containing TNFα (50 ng/mL; PeproTech, Rocky Hill, NJ, United States) (to generate MSC) or serum-free medium alone (to generate CM). In order to neutralize IL1RA in ^TNFα^CM, IL1RA antibody (2 μg/mL; R&D system, Minneapolis, MN, United States) was added to ^TNFα^CM and incubated for 1 h at 37°C. All CM were harvested, centrifuged for 10 min at 230 *g* to remove debris, and stored in 2 ml aliquots at −80°C until use.

### Detection of Cytokine

The levels of IL1RA were measured in the cell-free supernatants of MSCs using a rat IL1RA enzyme-linked immunosorbent assay (ELISA) kit (12 pg/mL detection limits) purchased from RayBiotech (Atlanta, GA, United States). The levels of IL10 and TNFα in the cell-free supernatants were determined by using ELISA (cat: ERC004; ERC102a), according to the experimental manual of Neobioscience Co. (Shenzheng, China).

### Statistical Analysis

For the mobilization experiment, we used at least five rats, and the experiments were performed in triplicate. *In vitro* cell culture experiments were performed in a non-blinded fashion, there was no randomization, and none of the samples was excluded from the analysis. Statistical analysis was performed using the SPSS 13.0 (SAS Institute, Chicago, IL, United States) for Windows. The distribution of variables was tested for normality using the Kolmogorov–Smirnov test. Comparisons between two groups were performed using independent unpaired two-tailed Student’s *t*-tests. Comparisons between multiple groups were performed using one-way analysis of variance with the Bonferroni correction for normally distributed data, and a non-parametric test (Kruskal–Wallis test) was used for non-normally distributed data. A *p* < 0.05 denoted statistical significance.

## Results

### The Traits of PB-MSCs and BM-MSCs

**FIGURE 1:**
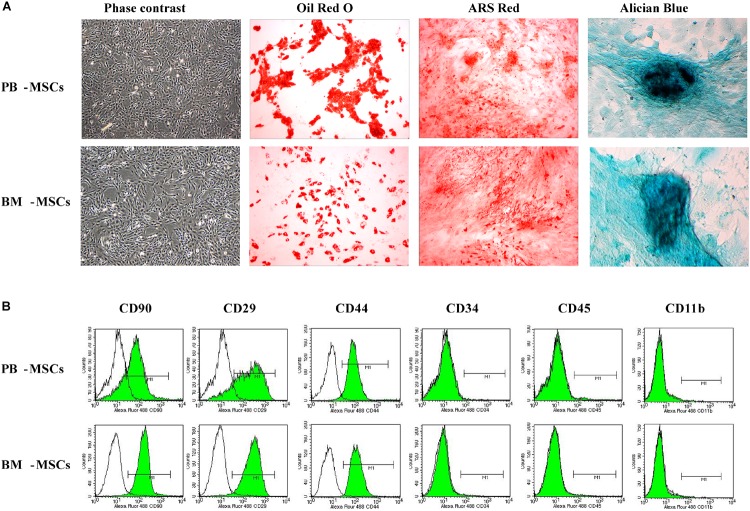
The morphologic and phenotypic characteristics and multiple-differentiation potential of BM-MSCs and PB-MSCs. **(A)** The typical image of third generation PB-MSCs and BM-MSCs with fibroblast-like shape. Osteogenesis of PB-MSCs and BM-MSCs was determined by Alizarin Red staining 21 days after osteogenic induction. Adipogenesis of PB-MSCs and BM-MSCs was detected via the formation of neutral lipid vacuoles stainable with Oil Red O staining 21 days after induction. Chondrogenesis of PB-MSCs and BM-MSCs was evaluated through Alician Blue staining 21 days after induction. **(B)** BM-MSCs and PB-MSCs were positive for CD44, CD29, and CD90, and negative for CD34, CD11b, and CD45.

We observed purified PB-MSCs and BM-MSCs (passaged thrice) under an inverted phase-contrast microscope to determine whether cultured PB-MSCs and BM-MSCs exhibited traits of stem cells. The investigation revealed that both types of cells were characterized by a fibroblast-like spindle shape (Figure 1A). In addition, the multi-differentiation potential of PB-MSCs and BM-MSCs was assayed. Both types of cells showed similar osteogenesis, adipogenesis, and chondrogenesis following induction with the corresponding protocols, as shown in Figure 1A. Red lipid droplets, red phosphate crystals, and blue nodules were observed in both differentiated PB-MSCs and BM-MSCs. Moreover, the immune phenotypes of PB-MSCs were consistent with those of BM-MSCs, along with positive expression for CD29, CD90, and CD44, and negative expression for CD11b, CD45, and CD34 (Figure 1B). These results suggested that the cultured PB-MSCs were MSCs with similar traits to those observed in BM-MSCs.

### PB-MSCs Efficiently Induced M0-Type Polarization Toward M2 Type

**FIGURE 2:**
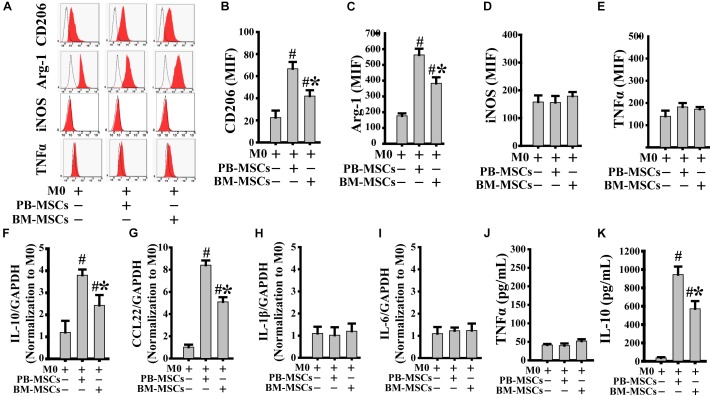
PB-MSCs efficiently promoted M0-type polarization toward M2 type. **(A–E)** M1 (i.e., iNOS and TNFα) or M2 (i.e., CD206 and Arg-1) type typical markers were evaluated by flow cytometry in M0-type macrophages following co-culture with either PB-MSCs or BM-MSCs using the Transwell system for 3 days. **(F–I)** The expression of pro-inflammatory (e.g., IL-6 and IL-1β) and anti-inflammatory (e.g., IL-10 and CCL-22) factors was determined by using a real-time PCR in M0 macrophage co-cultured with either PB-MSCs or BM-MSCs for 3 days. **(J,K)** The levels of pro-inflammatory cytokine (e.g., TNFα) and anti-inflammatory cytokine (e.g., IL-10) factors were detected by using ELISA in supernatants of M0 macrophages co-cultured with either PB-MSCs or BM-MSCs for 3 days. *n* = 5, *^#^p* < 0.05, vs. M0 co-cultured without either PB-MSCs or BM-MSCs, ^∗^*p* < 0.01, vs. M0 co-cultured with either PB-MSCs.

We assessed the typical markers of both M2 (CD206 and Arg-1) and M1 (iNOS and TNFα) to confirm whether co-culture with PB-MSCs or BM-MSCs affected M0 polarization toward either the M1 or M2 phenotype. As shown in Figure 2A–E, both PB-MSCs and BM-MSCs increased the expression levels of CD206 and Arg-1, indicating macrophage polarization from M0 to M2. Furthermore, M0 macrophages co-cultured with PB-MSCs showed greater generation of CD206- and Arg-1-positive M2 macrophages versus those co-cultured with BM-MSCs. Moreover, we detected IL-1β and IL-6 (typical pro-inflammatory factors), and IL-10 and CCL22 (typical anti-inflammatory factors) using real-time PCR analysis of macrophages to further confirm the functional traits of macrophages following co-culture with either PB-MSCs or BM-MSCs. As shown in Figure 2F–I, the expression levels of IL-1β and IL-6 were not different in response to M0 co-culture with PB-MSCs or BM-MSCs. Notably, the levels of IL-10 and CCL22 were markedly increased in response to co-culture of M0 with PB-MSCs or BM-MSCs. Furthermore, M0 macrophages co-cultured with PB-MSCs showed the greater expression of IL-10 and CCL22 versus those co-cultured with BM-MSCs. Moreover, the levels of IL10 in supernatants from the release of M0 macrophages co-cultured with PB-MSCs were higher than those co-cultured with BM-MSCs (Figure 2J,K). Thus, PB-MSCs more efficiently promoted M0 polarization toward M2 than BM-MSCs.

### PB-MSCs Triggered M1-Type Polarization Toward M2 Type

**FIGURE 3:**
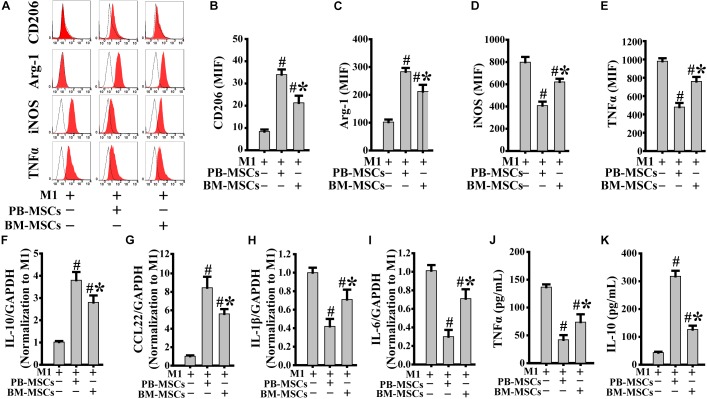
PB-MSCs triggered M1-type polarization toward M2 type. **(A–E)** PB-MSCs more effectively increased the expression of CD206 and Arg-1 in LPS-induced M1-type macrophages co-cultured with BM-MSCs using the Transwell system for 3 days, as determined by flow cytometry for M1 (i.e., iNOS and TNFα) or M2 (i.e., CD206 and Arg-1) typical markers. **(F–I)** The expression of pro-inflammatory (e.g., IL-6 and IL-1β) and anti-inflammatory (e.g., IL-10 and CCL-22) factors was determined using a real-time PCR in M1 macrophages co-cultured with either PB-MSCs or BM-MSCs for 3 days. **(J,K)** The levels of pro-inflammatory cytokine (e.g., TNFα) and anti-inflammatory cytokine (e.g., IL-10) factors were determined by using ELISA in supernatants of M1-type macrophages co-cultured with either PB-MSCs or BM-MSCs for 3 days. *n* = 5, *^#^p* < 0.05, vs. M1 co-cultured without either PB-MSCs or BM-MSCs, ^∗^*p* < 0.01, vs. M1 co-cultured with either PB-MSCs.

Macrophages release a large number of inflammatory factors that cause tissue damage related to M1 in response to inflammatory action (Novak and Koh, 2013). Therefore, modification of pro-inflammatory M1 into anti-inflammatory M2 is a crucial strategy in the treatment of inflammatory diseases. As shown in Figure 3A–E, PB-MSCs and BM-MSCs strongly increased the expression levels of CD206 and Arg-1, indicating macrophage polarization from M1 to M2. Furthermore, M1 macrophages co-cultured with PB-MSCs showed greater generation of CD206 and Arg-1-positive M2 macrophages compared with those co-cultured with BM-MSCs, indicating that PB-MSCs induced the lipopolysaccharide-induced M1 phenotypic switch toward M2. Consistent with the macrophage phenotypic switch from M1 to M2, the expression levels of inflammatory factors, including IL-1β and IL-6, were significantly decreased in response to M1 co-culture with PB-MSCs or BM-MSCs. Meanwhile, anti-inflammatory factors, such as IL-10 and CCL22, were markedly increased in response to M1 co-culture with PB-MSCs or BM-MSCs (Figure 3F–I). Importantly, M1 macrophages co-cultured with PB-MSCs showed higher expression of IL-10 and CCL22 and lower expression of IL-1β and IL-6 compared with those reported in cells co-cultured with BM-MSCs. Similarly, higher levels of IL10 and lower levels of TNFα were found in supernatants released from M1 macrophages co-cultured with PB-MSCs compared with those co-cultured with BM-MSCs (Figure 3J,K). Thus, PB-MSCs have a greater capability to modify M1 polarization toward M2, indicating that PB-MSCs play a more important anti-inflammatory role in the inflammatory microenvironment than BM-MSCs.

### PB-MSCs Exhibited a Stronger Anti-inflammatory Cytokine Expression Pattern

**FIGURE 4:**
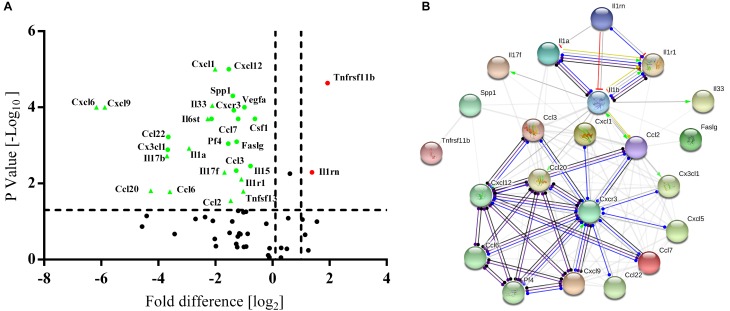
Differences between PB-MSCs and BM-MSCs analyzed using an inflammatory Cytokines and Receptors RT^2^ Profiler PCR Array. **(A)** The expressions of interleukin 1 receptor antagonist (IL1rn) and tumor necrosis factor receptor superfamily member 11b (Tnfrsf11b) genes were upregulated (fold change > 2.5, *p* < 0.001) while the 22 genes’ expressions were downregulated as analyzed by the PCR array. **(B)** The analysis of the gene network was performed using the STRING database.

High-throughput quantitative PCR arrays were used to detect the inflammatory factors expressed by BM-MSCs and PB-MSCs to evaluate whether the anti-inflammatory effects of PB-MSCs or BM-MSCs were associated with differences in inflammatory cytokines. As shown in Figure 4A, 24 differentially expressed genes were screened, including 22 downregulated and two upregulated genes. Interestingly, according to the STRING database, IL1rn may play a key role in the regulation of inflammatory factors targeting the differential gene interaction network (Figure 4B). These results suggested that the anti-inflammatory effects of PB-MSCs were mainly attributed to the release of anti-inflammatory factors.

### PB-MSC-Released IL1RA Was Involved in the Phenotypic Switch of M1 Toward M2

**FIGURE 5:**
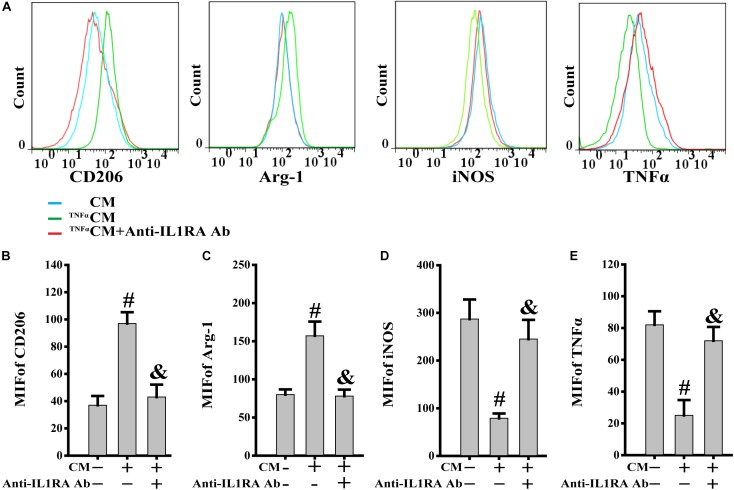
PB-MSCs-released IL1RA was involved in the M1-type switch into M2 type. **(A)** The typical results of M1 (i.e., iNOS and TNFα) or M2 (i.e., CD206 and Arg-1) type by flow cytometry in M1 macrophages treated with condition medium (CM) from TNFα-stimulated PB-MSCs (^TNFα^CM) with or without the presence of a neutralizing anti-IL1RA antibody for 3 days. **(B–E)** Quantitative analysis of results in **A** showed that CM promoted the polarization of the M1 type toward M2 type, which could be almost completely abolished by the anti-IL1RA antibody. *n* = 5, *^#^p* < 0.05, vs. CM, ^&^*p* < 0.01, vs. ^TNFα^CM.

TNFα, acting as a typical pro-inflammatory cytokine, was used to mimic the stimulation of inflammatory cytokines in PB-MSCs *in vivo* (Zhou et al., 2014). This analysis was performed to investigate the response of PB-MSCs to an inflammatory environment. As shown in Figure 5, the CM from TNFα-stimulated PB-MSCs (^TNFα^CM) promoted the expression of CD206 and Arg-1 (M2 markers), whereas it reduced that of iNOS and TNFα (M1 markers). These specific effects were abolished using the recombinant neutralizing anti-IL1RA antibody, indicating that PB-MSCs-released IL1RA was involved in the M1 switch to M2, in response to an inflammatory environment.

### Increased IL1RA Expression in PB-MSCs by TNFα Was Involved in NF-κB Signaling Pathway

**FIGURE 6:**
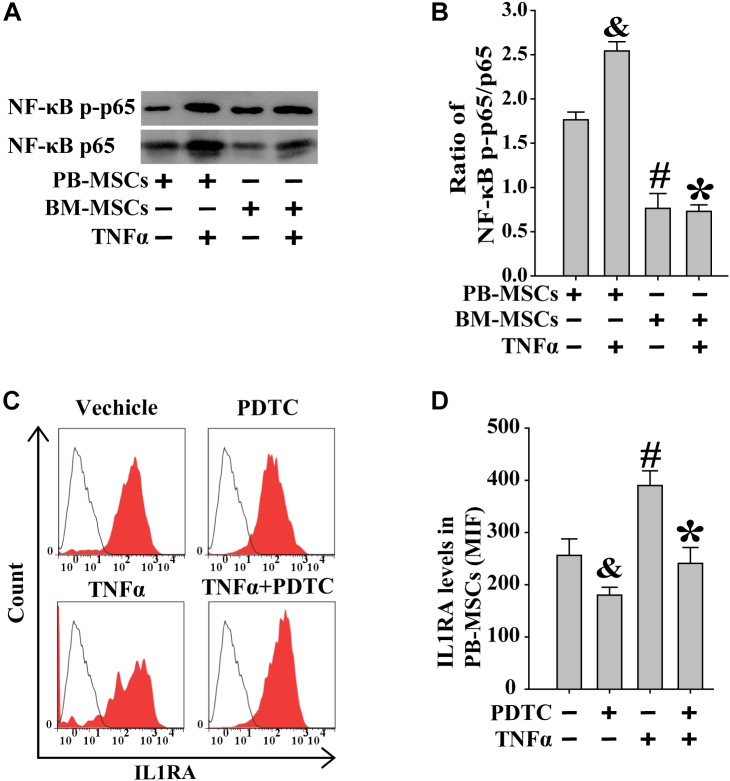
Increased IL1RA expression in PB-MSCs by TNFα was involved in NF-κB signaling pathway. **(A)** TNFα (50 ng/mL) increased the levels of NF-κB p-p65 and p65 in both BM-MSCs and PB-MSCs as detected by Western blot, especially in PB-MSCs. **(B)** Semi-quantitative analysis of results in **A** showed a higher ratio of NF-κB p-p65/p65 in PB-MSCs treated with TNFα (50 ng/ml) compared to BM-MSCs. *n* = 5, ^&^*p* < 0.05, vs. PB-MSCs; *^#^p* < 0.05, vs. PB-MSCs; ^∗^*p* < 0.05, vs. BM-MSCs. **(C)** Typical results of IL1RA expression in PB-MSCs as determined by flow cytometry. **(D)** Semi-quantitative analysis of results in **C** showed that TNFα (50 ng/mL) induced the expression of IL1RA in PB-MSCs, which could be obviously abolished by NF-κB inhibitor PDTC (100 μM). *n* = 5, ^&^*p* < 0.05, vs. PB-MSCs treated without TNFα and PDTC;*^#^p* < 0.05, vs. PB-MSCs treated without TNFα and PDTC; ^∗^*p* < 0.05, vs. BM-MSCs treated with TNFα.

Previous studies showed that exogenous TNFα may activate the NF-κB signaling pathway (Luz-Crawford et al., 2016). BM-MSCs and PB-MSCs were treated with TNFα to examine its role in NF-κB signaling in these cells. As shown in Figure 6A,B, following stimulation with TNFα, PB-MSCs showed higher levels of NF-κB compared with those observed in BM-MSCs. Subsequently, the expression of IL1RA in PB-MSCs, in response to stimulation with TNFα, was evaluated through fluorescence-activated cell sorting analysis. As shown in Figure 6C,D, TNFα promoted the expression of IL1RA in PB-MSCs, and these specific effects were abolished using the NF-κB inhibitor PDTC. These findings indicated that PB-MSCs stimulated by TNFα increased the expression of IL1RA.

### BM-MSCs Stimulated by Combination of G-CSF and AMD3100 Showed Higher Potential for Migration

**FIGURE 7:**
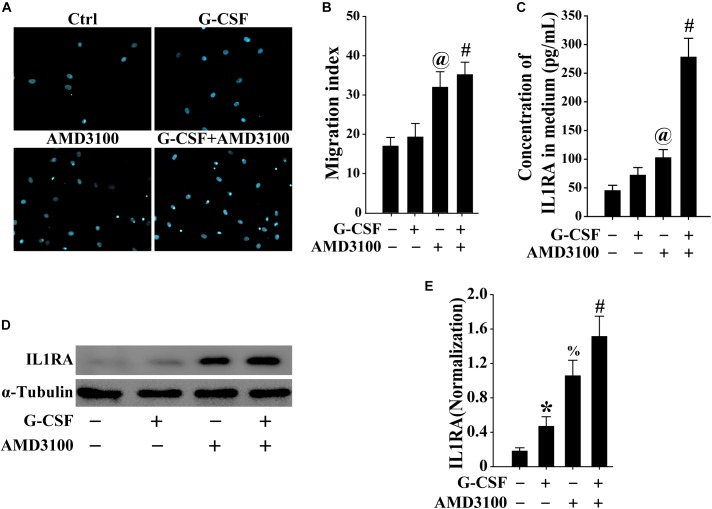
BM-MSCs treated with G-CSF/AMD3100 showed stronger migration potential. **(A)** Typical image of BM-MSCs migration treated with G-CSF and/or AMD3100 using the Transwell system. **(B)** Combined treatment of AMD3100 and G-CSF promoted greater BM-MSCs migration than single treatment of AMD3100 or G-CSF. *n* = 5, ^@^*p* < 0.05, vs. BM-MSCs treated with or without G-CSF; ^#^*p* < 0.05, vs. BM-MSCs treated with G-CSF. **(C)** Combined treatment of AMD3100 and G-CSF induced IL-1RA expression in BM-MSCs as detected by ELISA. *n* = 3, ^@^*p* < 0.05, vs. BM-MSCs treated with or without G-CSF; ^#^*p* < 0.05, vs. BM-MSCs treated with either G-CSF or AMD3100. **(D,E)** Combined treatment of AMD3100 and G-CSF increased IL-1RA protein levels in BM-MSCs as analyzed by Western blot **(D)** and semi-quantitative analysis **(E)**. *n* = 3, ^∗^*p* < 0.05, vs. BM-MSCs treated without either AMD3100 or G-CSF; ^%^*p* < 0.05, vs. BM-MSCs treated without either AMD3100 or G-CSF; ^#^*p* < 0.05, vs. BM-MSCs treated with either AMD3100 or G-CSF.

To mimic the mobilization of BM-MSCs into the peripheral blood, the migration of BM-MSCs was evaluated using the Transwell migration system. As shown in Figure 7A,B, both G-CSF and AMD3100 promoted the migration of BM-MSCs, especially their combination, indicating that more BM-MSCs may be mobilized from the bone marrow into the peripheral blood.

### Induced IL1RA Expression in BM-MSCs by Combination of G-CSF and AMD3100 Was Involved in NF-κB Signaling Pathway

As shown in Figure 7C,D, the expression of IL-1RA in BM-MSCs treated with either AMD3100 or G-CSF was higher compared with that observed in BM-MSCs. Moreover, the combination of AMD3100 and G-CSF resulted in the greatest induction of IL1rn in BM-MSCs, compared with AMD3100 or G-CSF alone. Furthermore, AMD3100 and/or G-CSF, especially their combination, induced the release of IL1RA in BM-MSCs (Figure 7E). These results indicated that AMD3100/G-CSF-treated BM-MSCs *in vitro* exhibit similar features to PB-MSCs mobilized by AMD3100/G-CSF.

**FIGURE 8:**
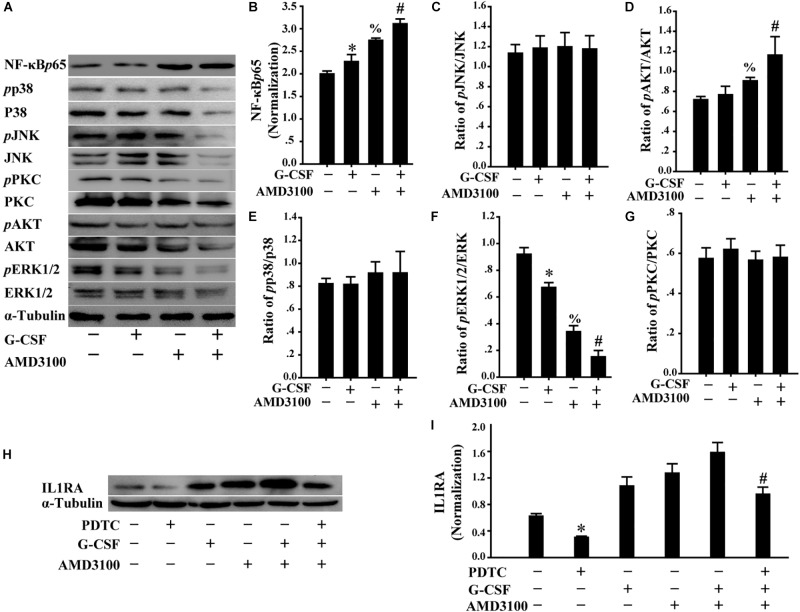
Induced IL1RA expression in BM-MSCs by a combination of G-CSF and AMD3100 was involved in NF-κB signaling pathway. **(A)** Western blot was used to detect the indicated proteins in BM-MSCs treated with G-CSF/AMD3100. **(B–G)** Semi-quantitative analysis of results in **A** showed higher levels of NF-κB protein and IL-1RA in BM-MSCs following the combined treatment of AMD3100 and G-CSF. *n* = 3, ^∗^*p* < 0.05, vs. BM-MSCs treated without either AMD3100 or G-CSF; ^%^*p* < 0.05, vs. BM-MSCs treated without either AMD3100 or G-CSF; ^#^*p* < 0.05, vs. BM-MSCs treated with either AMD3100 or G-CSF. **(H,I)** NF-κB inhibitor PDTC (100 μM) abolished the effects of AMD3100 and G-CSF on inducing IL-1RA expression in BM-MSCs as determined by Western blot **(H)** and semi-quantitative assay **(I)**. *n* = 3, ^∗^*p* < 0.05, vs. BM-MSCs treated without either AMD3100 or G-CSF; *^#^p* < 0.05, vs. BM-MSCs treated with combination of AMD3100 and G-CSF.

Furthermore, we analyzed the levels of multiple signal molecules, including NF-κB-p65, p38, JNK, PKC, AKT, and ERK using Western blot to investigate the potential signal mechanism of IL1RA expression in BM-MSCs following stimulation with AMD3100/G-CSF. As shown in Figure 8A–G, in BM-MSCs, the combination of AMD3100 and G-CSF markedly increased the level of NF-κB-p65 protein compared with that observed in cells treated with AMD3100 and G-CSF alone. Importantly, AMD3100/G-CSF-induced expression of IL1RA may be markedly abolished using the NF-κB inhibitor PDTC (Figure 8H,I). These results indicate that the expression of IL1RA induced by AMD3100/G-CSF in BM-MSCs involves the NF-κB signaling pathway.

## Discussion

The present study offers three novel observations. First, PB-MSCs mobilized by AMD3100/G-CSF played a greater role in inducing the phenotypic switch from M0 or M1 to M2 versus BM-MSCs. Second, PB-MSCs demonstrated stronger activation of NF-κb and greater expression and release of IL1RA, leading to greater M2 polarization of macrophages in response to an inflammatory environment (i.e., TNFα). Lastly, AMD3100/G-CSF-treated BM-MSCs *in vitro* showed similar characteristics to those of PB-MSCs in terms of IL1rn expression and release, which were associated with the NF-κB signaling pathway.

Macrophage activation and phenotypic switch from macrophages (M0) to a pro-inflammatory phenotype (M1) and anti-inflammatory phenotype (M2) are involved in the inflammatory response and development of various human diseases, such as heart disease, diabetes mellitus, and Alzheimer’s disease (Novak and Koh, 2013). MSCs are involved in the regeneration and repair of tissues and organs due to their multi-differentiation potential (Wong, 2011). These cells have shown attractive and unique effects on inflammatory modulation (Machado et al., 2013; Wang et al., 2014), inducing either M0 or M1 macrophage polarization toward M2 (Kim and Hematti, 2009; Nemeth et al., 2009; Chiossone et al., 2016; Luz-Crawford et al., 2016). Thus, MSC therapy may be a promising strategy for the treatment of inflammatory diseases.

Although PB-MSCs originated from the mobilization of BM-MSCs, the differences between these two types of cells in inflammatory regulation remain unclear (Ok et al., 2015). In this study, using co-culture of MSCs with macrophages mediated through the Transwell system, PB-MSCs mobilized by AMD3100/G-CSF greatly promoted M0 or M1 macrophage polarization toward M2 macrophages versus BM-MSCs. This finding was consistent with those reported in previous studies (Machado et al., 2013; Zhou et al., 2013). The present study demonstrated evidence that the significant differences in IL1rn between BM-MSCs and PB-MSCs contributed to the induction of M2 polarization in macrophages.

Recent evidence from MSC transplantation studies supports the concept that MSC-mediated inflammatory modulation depends on the interaction between the transplanted MSCs and the microenvironment (Wang et al., 2016). TNFα is a typical pro-inflammatory factor within the inflammatory microenvironment (Vaz et al., 2011). In the present study, using TNFα to mimic the inflammatory microenvironment, we found that PB-MSCs showed higher expression and release of IL1RA in response to stimulation by TNFα. Further findings showed that TNFα activated NF-κb signaling in PB-MSCs, increasing the level of IL1RA in PB-MSCs. Furthermore, the NF-κb inhibitor PDTC markedly reduced the expression and secretion of IL1RA in PB-MSCs after stimulation by TNFα. More importantly, the effects of ^TNFα^CM on M1 polarization toward M2 were abolished using a neutralizing IL1RA antibody. Thus, PB-MSCs within the inflammatory microenvironment may act as an anti-inflammatory player through the TNFα-NF-κb-IL1RA integral system between PB-MSCs and macrophages.

To explore possible alternative cells due to the problem of PB-MSCs, culture *in vitro* for large-scale clinical use compared with BM-MSCs which are easier to cultivate. In the present study, AMD3100/G-CSF-treated BM-MSCs and PB-MSCs showed increased expression and release of IL1RA. The differences between AMD3100/G-CSF-treated BM-MSCs and AMD3100/G-CSF-mobilized PB-MSCs will be further investigated in future studies. Nevertheless, AMD3100/G-CSF-treated BM-MSCs may be a novel agent for the regeneration and repair of tissues and organs, especially in inflammatory diseases.

## Conclusion

**FIGURE 9:**
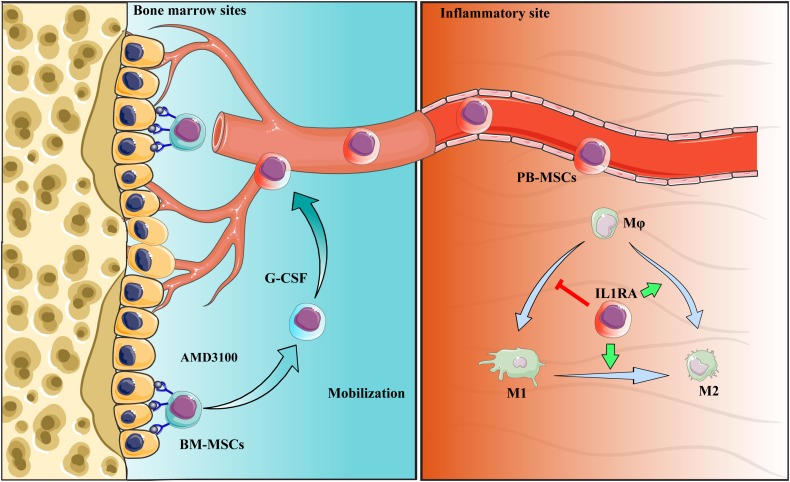
Schematic diagram of the interaction between PB-MSCs and macrophages. Quiescent MSCs inhabit the hematopoietic niche, which is mainly composed of osteoblasts and the vascular network. In the process of mobilization by G-CSF combined with AMD3100, BM-MSCs detached from the niche because of block of SDF-1α/CXCR4 axis by AMD3100 to cut off SDF-1α binding into CXCR4, and G-CSF to reduce SDF-1α levels in the niche (Petit et al., 2002; Tang et al., 2011; Hoggatt et al., 2018). Mobilized BM-MSCs enter peripheral blood, functioning as PB-MSCs. PB-MSCs contributed to the polarization of M1 toward M2 type in the inflammatory sites though IL1RA, which were associated with NF-κB signaling pathway.

Collectively, PB-MSCs mobilized by G-CSF/AMD3100 exhibited stronger immunomodulatory functions in promoting M2 polarization of macrophages versus BM-MSCs. These anti-inflammatory effects were attributed to the activation of the NF-κB-IL1RA signaling pathway. By targeting IL1rn, gene modification or drug intervention of MSCs may provide a novel therapeutic strategy against human diseases, especially inflammatory diseases (Figure 9).

## Ethics Statement

This study was carried out in accordance with the recommendations of the Laboratory Animal Centre, Hubei University of Medicine. All animal experiments protocols were approved by the Hubei University of Medicine Health Network Animal Care Committees (SCXK 2011-0008).

## Author Contributions

LC carried out main cell experiments and drafted the manuscript. F-YR, XL, QF, and G-YS carried out qPCR, protein detection, and immunostaining. LC, J-MT, QZ, Q-HC, L-MY, and TZ conceived of the study, participated in the experimental design and coordination of the study, and helped to draft the manuscript. All authors read and approved the final manuscript.

## Conflict of Interest Statement

The authors declare that the research was conducted in the absence of any commercial or financial relationships that could be construed as a potential conflict of interest.
