# Supplementary material for: Combination of G-CSF and AMD3100 Improves the Anti-inflammatory Effect of Mesenchymal Stem Cells on Inducing M2 Polarization of Macrophages Through NF-κB-IL1RA Signaling Pathway
Source: Front Pharmacol. 2019 May 28;10:579. doi: 10.3389/fphar.2019.00579 (PMC6546872; doi:10.3389/fphar.2019.00579)
Supplement: Supplementary file 1 [file Table_1.docx]

**Additional files**

Combination of G-CSF and AMD3100 improve the anti-inflammatory effect of mesenchymal stem cells on inducing M2 polarization of macrophages through NF-κB-IL1RA signaling pathway

Long Chen^1, 2^, Qian Zhang^4^, Qin-Hua Chen^2^, Feng-Yin Ran^2^, Li-Mei Yu^1^, Xiu Liu^1^, Qiang Fu^1^, Gong-Yu Song^4^, Jun-Ming Tang^3, 5*^, Tao Zhang ^1*^

^1^ Key Laboratory of Cell Engineering of Guizhou Province, Affiliated Hospital of Zunyi Medical University, Zunyi, China;

^2^ Experimental Medical Center, Dongfeng Hospital, Hubei University of Medcine, Shiyan, China;

^3^Institute of Clinical Medicine, Renming Hospital, Hubei University of Medicine, Shiyan, China;

^4^ Department of Human Anatomy, Zunyi Medical University, Zunyi, China.

^5^ Institute of biomedicine and Key Lab of Human Embryonic Stem Cell of Hubei Province, Hubei University of Medicine, Hubei 442000, China

**Correspondence author:**

Tao Zhang, Key Laboratory of Cell Engineering of Guizhou Province, Affiliated Hospital of Zunyi Medical university, 149 Dalian Rd., Zunyi 563000, China. Tel.: 0086-851-28608806; E-mail: [oceanzt@163.com](mailto:oceanzt@163.com);

**Co-correspondence author:**

Jun-ming Tang, Institute of Clinical Medicine, Renming Hospital, Hubei University of Medicine, Shiyan, 442000, China. Tel.: 0719-8637791; E-mail: [tangjm416@163.com](mailto:tangjm416@163.com).

**Supplemental Table 1. PCR-ARRAY information**

| **Symbol** | **Description** | **GenBank** |
| --- | --- | --- |
| Aimp1 | Aminoacyl tRNA synthetase complex-interacting multifunctional protein 1 | NM_053757 |
| Bmp2 | Bone morphogenetic protein 2 | NM_017178 |
| Ccl11 | Chemokine (C-C motif) ligand 11 | NM_019205 |
| Ccl12 | Chemokine (C-C motif) ligand 12 | NM_001105822 |
| Ccl17 | Chemokine (C-C motif) ligand 17 | NM_057151 |
| Ccl19 | Chemokine (C-C motif) ligand 19 | NM_001108661 |
| Ccl2 | Chemokine (C-C motif) ligand 2 | NM_031530 |
| Ccl20 | Chemokine (C-C motif) ligand 20 | NM_019233 |
| Ccl22 | Chemokine (C-C motif) ligand 22 | NM_057203 |
| Ccl24 | Chemokine (C-C motif) ligand 24 | NM_001013045 |
| Ccl3 | Chemokine (C-C motif) ligand 3 | NM_013025 |
| Ccl4 | Chemokine (C-C motif) ligand 4 | NM_053858 |
| Ccl5 | Chemokine (C-C motif) ligand 5 | NM_031116 |
| Ccl6 | Chemokine (C-C motif) ligand 6 | NM_001004202 |
| Ccl7 | Chemokine (C-C motif) ligand 7 | NM_001007612 |
| Ccl9 | Chemokine (C-C motif) ligand 9 | NM_001012357 |
| Ccr1 | Chemokine (C-C motif) receptor 1 | NM_020542 |
| Ccr10 | Chemokine (C-C motif) receptor 10 | NM_001108836 |
| Ccr2 | Chemokine (C-C motif) receptor 2 | NM_021866 |
| Ccr3 | Chemokine (C-C motif) receptor 3 | NM_053958 |
| Ccr4 | Chemokine (C-C motif) receptor 4 | NM_133532 |
| Ccr5 | Chemokine (C-C motif) receptor 5 | NM_053960 |
| Ccr6 | Chemokine (C-C motif) receptor 6 | NM_001013145 |
| Ccr8 | Chemokine (C-C motif) receptor 8 | XM_236704 |
| Cd40lg | CD40 ligand | NM_053353 |
| Csf1 | Colony stimulating factor 1 (macrophage) | NM_023981 |
| Csf2 | Colony stimulating factor 2 (granulocyte-macrophage) | XM_340799 |
| Csf3 | Colony stimulating factor 3 (granulocyte) | NM_017104 |
| Cx3cl1 | Chemokine (C-X3-C motif) ligand 1 | NM_134455 |
| Cx3cr1 | Chemokine (C-X3-C motif) receptor 1 | NM_133534 |
| Cxcl1 | Chemokine (C-X-C motif) ligand 1 (melanoma growth stimulating activity, alpha) | NM_030845 |
| Cxcl10 | Chemokine (C-X-C motif) ligand 10 | NM_139089 |
| Cxcl11 | Chemokine (C-X-C motif) ligand 11 | NM_182952 |
| Cxcl12 | Chemokine (C-X-C motif) ligand 12 (stromal cell-derived factor 1) | NM_022177 |
| Cxcl2 | Chemokine (C-X-C motif) ligand 2 | NM_053647 |
| Cxcl5 | Chemokine (C-X-C motif) ligand 5 | NM_022214 |
| Cxcl9 | Chemokine (C-X-C motif) ligand 9 | NM_145672 |
| Cxcr2 | Chemokine (C-X-C motif) receptor 2 | NM_017183 |
| Cxcr3 | Chemokine (C-X-C motif) receptor 3 | NM_053415 |
| Cxcr5 | Chemokine (C-X-C motif) receptor 5 | NM_053303 |
| Faslg | Fas ligand (TNF superfamily, member 6) | NM_012908 |
| Ifng | Interferon gamma | NM_138880 |
| Il10ra | Interleukin 10 receptor, alpha | NM_057193 |
| Il11 | Interleukin 11 | NM_133519 |
| Il13 | Interleukin 13 | NM_053828 |
| Il15 | Interleukin 15 | NM_013129 |
| Il16 | Interleukin 16 | NM_001105749 |
| Il17a | Interleukin 17A | NM_001106897 |
| Il17b | Interleukin 17B | NM_053789 |
| Il17f | Interleukin 17F | NM_001015011 |
| Il1a | Interleukin 1 alpha | NM_017019 |
| Il1b | Interleukin 1 beta | NM_031512 |
| Il1r1 | Interleukin 1 receptor, type I | NM_013123 |
| Il1rn | Interleukin 1 receptor antagonist | NM_022194 |
| Il21 | Interleukin 21 | NM_001108943 |
| Il27 | Interleukin 27 | XM_344962 |
| Il2rb | Interleukin 2 receptor, beta | NM_013195 |
| Il2rg | Interleukin 2 receptor, gamma | NM_080889 |
| Il3 | Interleukin 3 | NM_031513 |
| Il33 | Interleukin 33 | NM_001014166 |
| Il4 | Interleukin 4 | NM_201270 |
| Il5 | Interleukin 5 | NM_021834 |
| Il5ra | Interleukin 5 receptor, alpha | NM_053645 |
| Il6r | Interleukin 6 receptor | NM_017020 |
| Il6st | Interleukin 6 signal transducer | NM_001008725 |
| Il7 | Interleukin 7 | NM_013110 |
| Il8ra | Interleukin 8 receptor, alpha | NM_019310 |
| Lta | Lymphotoxin alpha (TNF superfamily, member 1) | NM_080769 |
| Ltb | Lymphotoxin beta (TNF superfamily, member 3) | NM_212507 |
| Mif | Macrophage migration inhibitory factor | NM_031051 |
| Nampt | Nicotinamide phosphoribosyltransferase | NM_177928 |
| Osm | Oncostatin M | NM_001006961 |
| Pf4 | Platelet factor 4 | NM_001007729 |
| RGD1561905_predicted | Complement component 5 | XM_345342 |
| Spp1 | Secreted phosphoprotein 1 | NM_012881 |
| Tnf | Tumor necrosis factor (TNF superfamily, member 2) | NM_012675 |
| Tnfrsf11b | Tumor necrosis factor receptor superfamily, member 11b | NM_012870 |
| Tnfsf10 | Tumor necrosis factor (ligand) superfamily, member 10 | NM_145681 |
| Tnfsf11 | Tumor necrosis factor (ligand) superfamily, member 11 | NM_057149 |
| Tnfsf13 | Tumor necrosis factor (ligand) superfamily, member 13 | NM_001009623 |
| Tnfsf13b | Tumor necrosis factor (ligand) superfamily, member 13b | NM_001109112 |
| Tnfsf14 | Tumor necrosis factor (ligand) superfamily, member 14 | XM_236794 |
| Tnfsf4 | Tumor necrosis factor (ligand) superfamily, member 4 | NM_053552 |
| Vegfa | Vascular endothelial growth factor A | NM_031836 |
| Actb | Actin, beta | NM_031144 |
| B2m | Beta-2 microglobulin | NM_012512 |
|  |  |  |
|  |  |  |

Supplemental table 2. Primers for Real-Time PCR

| *Gene* | *Primer sequence* | *Annealing temperature (*°*C)* | *Cycles* | *Size (bp)* |
| --- | --- | --- | --- | --- |
| *IL-6* | *AGAGACTTCCAGCCAGTTGC* | *63* | *40* | *85* |
|  | *AGTCTCCTCTCCGGACTTGT* |  |  |  |
| *IL-10* | *GAAGCTGAAGACCCTCTGGATACA* | *60* | *40* | *117* |
|  | *CCTTTGTCTTGGAGCTTATTAAAATCA* |  |  |  |
| *IL1β* | *AATGACCTGTTCTTTGAGGCTGAC* | *60* | *40* | *115* |
|  | *CGAGATGCTGCTGTGAGATTTGAAG* |  |  |  |
| *CCL22* | *GGCAGGAAGGACCATACAAA* | *60* | *40* | *200* |
|  | *TCCAGAGGAGCAAGCAGATT* |  |  |  |
| *GAPDH* | *ATGCTGGTGCTGAGTATGTC* | *60* | *30* | *162* |
|  | *AGTTGTCATATTTCTCGTGG* |  |  |  |

**Supplemental Table 3. Primary antibodies used for Western blot**

| **Rat antigens** | **Poly/mono- clonal** | **Manufacturer** | **Dilution** |
| --- | --- | --- | --- |
| IL1RA | monoclonal | Abcam, US.  Cat. No: ab124962 | 1:1000 for Western blot(1:50 for FCM) |
| Phospho-P38 MAPK | polyclonal | Arigo biolaboratories Corp, Taiwan  Cat. No: ARG51850 | 1:1000 for Western blot |
| P38 MAPK | polyclonal | GeneTex, US  Cat. No: GTX110720 | 1:1000 for Western blot |
| Phospho-ERK1/2 | polyclonal | R&D systems , US  Cat. No: AF1018 | 1:1000 for Western blot |
| ERK1/2 | polyclonal | Proteintech ,US  Cat. No: 16443-1AP | 1:1000 for Western blot |
| Phospho-SAPK/JNK | polyclonal | Arigo biolaboratories Corp, Taiwan  Cat. No: ARG51807 | 1:1000 for Western blot |
| JNK | monoclonal | Abcam, US  Cat. No: ab179461 | 1:1000 for Western blot |
| Phospho-AKT | polyclonal | GeneTex, US  Cat. No: GTX128414 | 1:1000 for Western blot |
| AKT | polyclonal | GeneTex, US  Cat. No: GTX121937 | 1:500 for Western blot |
| Phospho-PKC | polyclonal | GeneTex, US  Cat. No: GTX130453 | 1:1000 for Western blot |
| PKC | polyclonal | GeneTex, US  Cat. No: GTX133936 | 1:1000 for Western blot |
| NF-κb p65 | polyclonal | GeneTex, US  Cat. No: GTX102090 | 1:1000 for Western blot |
| NF-κb p65  (phospho S536) | polyclonal | Abcam, US.  Cat. No: ab86299 | 1:1000 for Western blot |
| α-tubulin | monoclonal | Sigma-Aldrich, US  Cat. No: T6199 | 1:10000 for Western blot |
